# Comparing Gamma Knife and CyberKnife in patients with brain metastases

**DOI:** 10.1120/jacmp.v15i1.4095

**Published:** 2014-01-06

**Authors:** Terence T. Sio, Sunyoung Jang, Sung‐Woo Lee, Bruce Curran, Anil P. Pyakuryal, Edward S. Sternick

**Affiliations:** ^1^ Department of Radiation Oncology Mayo Clinic Rochester MN; ^2^ Princeton Radiation Oncology Monroe NJ; ^3^ Department of Radiation Oncology Rhode Island Hospital Providence RI; ^4^ Department of Physics University of Illinois at Chicago Chicago IL

**Keywords:** Gamma Knife, CyberKnife, dosimetry, dosimetric comparison, brain metastases

## Abstract

The authors compared the relative dosimetric merits of Gamma Knife (GK) and CyberKnife (CK) in 15 patients with 26 brain metastases. All patients were initially treated with the Leksell GK 4C. The same patients were used to generate comparative CK treatment plans. The tissue volume receiving more than 12 Gy (V12), the difference between V12 and tumor volume (V12net), homogeneity index (HI), and gradient indices (GI25, GI50) were calculated. Peripheral dose falloff and three conformity indices were compared. The median tumor volume was 2.50 cm^3^ (range, 0. 044‐19.9). A median dose of 18 Gy (range, 15‐22) was prescribed. In GK and CK plans, doses were prescribed to the 40‐50% and 77‐92% isodose lines, respectively. Comparing GK to CK, the respective parametric values (median±standard deviation) were: minimum dose (18.2±3.4 vs. 17.6±2.4 Gy, p=0.395); mean dose (29.6±5.1 vs.20.6±2.8 Gy, p<0.00001); maximum dose (40.3±6.5 vs.22.7±3.3 Gy, p<0.00001); and HI (2.22±0.19 vs. 1.18±0.06, p<0.00001). The median dosimetric indices (GK vs. CK, with range) were: RTOG_CI, 1.76 (1.12‐4.14) vs. 1.53 (1.16‐2.12), p=0.0220; CI, 1.76 (1.15‐4.14) vs. 1.55 (1.18‐2.21), p=0.050; nCI, 1.76 (1.59‐4.14) vs. 1.57 (1.20‐2.30), p=0.082; GI50, 2.91 (2.48‐3.67) vs. 4.90 (3.42‐11.68), p<0.00001; GI25, 6.58 (4.18‐10.20) vs. 14.85 (8.80‐48.37), p<0.00001. Average volume ratio (AVR) differences favored GK at multiple normalized isodose levels (p<0.00001). We concluded that in patients with brain metastases, CK and GK resulted in dosimetrically comparable plans that were nearly equivalent in several metrics, including target coverage and minimum dose within the target. Compared to GK, CK produced more homogenous plans with significantly lower mean and maximum doses, and achieved more conformal plans by RTOG_CI criteria. By GI and AVR analyses, GK plans had sharper peripheral dose falloff in most cases.

PACS number: 89.20.‐a

## I. INTRODUCTION

Brain metastases significantly shorten the lives of cancer patients, with the majority of primary tumors originating from lung, breast, skin (melanoma), kidney, and gastrointestinal organs. It represents a significant clinical burden, with an incidence of at least 40% in advanced‐stage cancer patients, and directly responsible for an estimated 20% of cancer deaths.^(1)^ Economically, brain metastases represent a significant burden in total health‐care expenditure for cancer‐related treatments.^(2)^

Brain metastases occur more commonly than primary brain tumors in adults.^(3)^ A metropolitan study reported that 19.9% of lung cancer patients developed brain metastases, followed by melanoma (6.9%), renal (6.5%), breast (5.1%), and colorectal cancer (1.8%).^(4)^ Stereotactic radiosurgery (SRS) is effective for palliating intracranial metastases, even from radio‐resistant tumors such as melanoma.^(5)^

Prognosis for patients with brain metastases remains very poor, typically with median survival ranges from 2.3‐7.1 months.^(6)^ Treatment options include expectant medical management, systemic chemotherapy, biological agents, surgery, whole‐brain radiotherapy (WBRT), and local boost with SRS.^(7)^ In patients with single brain metastasis, adding adjuvant WBRT after surgery decreased the rate of local recurrence.^(8)^ However, up to 10% of patients receiving WBRT may experience cognitive deterioration, short‐term memory loss, and radiation‐induced dementia.^(9)^ Increasingly, radiation oncologists and neurosurgeons prefer using local techniques, such as SRS and surgery, as first‐line treatments in patients with oligometastatic brain tumors, while deferring WBRT as a salvage option.

For patients with reasonable performance status and life expectancy, the American Society for Radiation Oncology (ASTRO) supports the use of WBRT with a radiosurgery boost to control up to four brain metastases. The combination of WBRT and SRS significantly improves survival in patients with single brain metastases.^(10)^ For selected patients with good performance status and limited metastatic burden, treatment with SRS alone is a reasonable option. Stereotactic boosts can be carried out in several modalities, such as Gamma Knife (GK) (Elekta AB, Stockholm, Sweden), CyberKnife (CK) (Accuray Inc., Sunnyvale, CA), and various linac‐based systems such as Novalis (BrainLAB, Feldkirchen, Germany). Other modalities include tomotherapy, proton radiotherapy, and volumetric‐arc modulated therapy can also deliver SRS. Regardless of modality choice, RTOG 9005 established dose escalation schedule for brain metastases, based on diameter.^(11)^ Doses vary from 15 to 24 Gy, and are inversely related to size (up to 40 mm) in order to minimize possibility of radiation necrosis. SRS also has a role in treatment of previously resected cavities of brain metastases.^(12)^

In this study, two common SRS modalities (GK and CK) will be dosimetrically compared. Gamma Knife is probably the most well‐known SRS system in the world. Brain metastases typically represent more than 50% of GK cases at any institution. The radiological concept of the GK system is fairly simple: it utilizes 201 concentrically placed Cobalt‐60 energy sources to concentrate beams from different angles into a precisely defined spot inside the skull. The patient's position is fixed by a rigid metal headframe, which allows for accuracy in beam delivery from many directions and a focused radiation dose.

CK first obtained their FDA approval for therapeutic use in humans in 2001. Since then, there has been an expanding use of this versatile system worldwide. CK utilizes 6 MV photon beams produced by a compact linear accelerator, which in turn is mounted on a robotic arm with six degrees of translational and rotational freedom for spatial beam introduction. Stereotactically, CK relies on new assistive and adaptive technology called image‐guided radiotherapy (IGRT) for tracking its target(s) both in space and time. Compared to GK, this technology allows CK to introduce a frameless treatment option for patients with brain metastases. CK delivers nonisocentric beams with a highly conformal dosing schedule and gives precision of beam delivery at submillimeter range by IGRT technologies.^(13)^

As CyberKnife is still a relatively new technology, few direct comparison studies with other SRS systems have been published in the literature.^(14)^ A recently published case‐controlled study reported a detailed dosimetric comparison between the two modalities in patients with single brain metastases, but their survival analysis was confounded due to the CK patients receiving more modern chemotherapies.^(15)^ In another retrospective series,^(16)^ 25 patients with brain metastases from non‐small cell lung cancer were either treated with GK or CK. A total of 56/58 (97%) lesions were successfully controlled.

## II. MATERIALS AND METHODS

The authors performed a head‐to‐head, quantitative comparison of dosimetric profiles between the Leksell Gamma Knife C and CyberKnife robotic radiosurgery systems. Dual treatment plans based on 15 patients with 26 existing brain metastases were created and compared according to dosimetric parameters and indices. Difference in conformity, dose homogeneity, and peripheral dose falloff was also evaluated. This study explored the relative merits of dosing capacities and capabilities between the two SRS systems.

**Figure 1:**
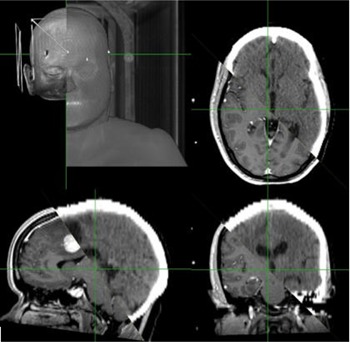
Overall, axial, coronal, and sagittal views (clockwise from top left) of CT/MR fusion in CK MultiPlan.

From 5/27/2008 to 2/3/2010, 15 patients previously treated on the Leksell Gamma Knife C radiosurgery system were selected. The Institutional Review Board (FWA‐00001230, IRB Registration #0004624) approved the study. These patients all had a deliverable GK plan produced by Leksell GammaPlan 8.3 (Elekta). The GK plans were generated according to an institutional protocol, with adherence to the RTOG guidelines and respecting critical organ constraints such as the optic chiasm and brain stem. For comparison, the Accuray treatment planning system MultiPlan DTS 3.0 was used for reproducing treatment plans previously delivered by the GK system. Identical stereotactic MRI images were transferred to CK, including weighted T2 & FLAIR sequences (5 mm thick), a T1‐weighted sequence (5 mm thick), and axial/coronal 3D‐MPRAGE sequences (2 mm thick). Computed tomography (CT) series was also acquired, which was fused with MR by manual seed point registration and algorithm‐assisted translational and rotational steps (see Fig. 1). After quality fusion images were created, critical organ structures (also called volume of interest (VOI)) including spinal cord, brainstem, eyes, lens, optic nerve tracts, optic chiasm, and pituitary gland, were outlined in axial CT/MR images. These critical organ constraints were respected and must be met in the dose optimization process. The gross tumor volume (GTV) (also a VOI) was designated as target which matched the GK's volume. The GTV's location, target size, shape, and convexity were well‐matched (<5% volume difference in all duplicated lesions). A clinical objective list and relaxed convergence values for each individual step were then decided and carried out in a temporal order as predefined by a user's script. Two additional hollow contour sets (shells), 3 mm and 30 mm away from the

GTV, were created to optimize the dose distribution to normal tissues. The prescription isodose percentage was applied to optimize the GTV coverage to 97%‐100%. The ray‐tracing algorithm generated an initial beam set and began the sequential optimization process. Identical marginal dose prescription was given in each pair of comparative CK and GK plans. A high‐resolution calculation step was performed in the evaluate step to finalize the CK treatment plan.

In both GK and CK systems, the minimum, mean, and maximum doses were calculated and compared. The homogeneity index (HI) measured as the ratio of maximum dose over prescription dose, was also reported. V12 was the tissue volume receiving at least 12 Gy, and V12net was the difference between V12 and tumor volume. GI50 was the ratio of prescription isodose volume (PIV) to the isodose volume receiving half of the prescription dose, which is a commonly used index in comparing various rival plans.^(17)^ PIV represents the three‐dimensional volume which receives the prescription dose or more, as enclosed by the prescription isodose contour at that level. Dose‐volume histogram (DVH) tables were extracted from both GK and CK planning software programs for peripheral dose falloff calculations. Conformity and homogeneity indices were calculated for all GK and CK plans. The Appendix summarizes the theory and dosimetric concepts employed in this study.

## III. RESULTS

Table 1 summarizes the patients’ demographics and tumor characteristics in this study. The median age was 63 years old. The primary tumor sites included lung (SCLC and NSCLC), breast, colorectal, skin (melanoma), and non‐Hodgkin's lymphoma. Most patients had an excellent Karnofsky Performance Score (KPS) at the time of receiving SRS (13 patients had documented KPS 80 or above, with one inpatient case having a KPS of 50). Five patients received prior surgical resections, with six resection cavities ranging 2.9‐19.9 cm^3^. Three patients (two received total gross resections, one subtotal resection) underwent GK as a postoperative boost, three to four weeks after their initial surgeries. Two other patients received salvage GK as local recurrence developed.

The 15 patients altogether presented with a total of 65 lesions, which were all treated by GK accordingly. Three patients had a total of 12, 12, and 9 lesions, respectively. Twenty‐six representative lesions (including all surgically resected cases) were then selected and replanned in CK. They were well‐distributed in both cerebral and cerebellar hemispheres, representing typical pattern of brain metastases (see Table 2). The excluded lesions were generally small (<1 cm in diameter) and of less dosimetric interest. Special cases included one lesion in the right internal auditory canal and another one in the brainstem. Most of these lesions resembled spheres in shape, except for the resected cavities which appeared more irregular. A balanced distribution of tumor sizes was achieved: <1 cm, 4 (15%); 1‐1.99 cm, 11 (42%); 2‐2.99 cm, 9 (35%), and 3‐4 cm, 2 (8%).

**Table 1:** Demographics in the GK and CK comparison study (15 patients)

	*n(%) or Median (range)*
Male	5 (33%)
Age	63 (30‐80)
Primary Site	
SCLC	1 (6.7%)
NSCLC	6 (40%)
Colorectal	2 (13%)
Breast	3 (20%)
Melanoma	2 (13%)
NHL	1 (6.7%)
WBRT prior to SRS	2 (13%)
Surgery prior to SRS	
Total resection	4 (27%)^a^
Subtotal resection	1 (6.7%)
Number of brain metastases	2 (1‐12)
Karnofsky performance score	90 (50‐100)

a ^a^ Two cases presented as recurrence, for salvage consideration with SRS.

GK=Gamma Knife; CK=CyberKnife; SCLC=small cell lung cancer; NSCLC=non−small cell lung cancer; $\text{NHL}={\text{non}-\text{Hodgkin}}^{'}\text{s lymphoma}$; WBRT=whole brain radiation therapy; SRS=stereotactic radiosurgery.

Basic parameters for both GK and CK dosimetric plans are shown in Table 3. All GammaPlan tumor volumes were reproduced well in CK's MultiPlan. The shape and position of these tumors were well‐preserved, with a pair‐wise volume difference of no more than 5%. Same prescription dose was given to each pair of plans. The median prescription dose was 18 Gy (range 15‐22 Gy), all delivered in 1 fraction.

Due to intrinsic difference between GK's isocentric and CK's nonisocentric planning, prescription isodose percentage must be altered when cases were re‐simulated in CK. In GK, radiation oncologists typically prescribe to an isodose line of 45%‐55% for brain metastases. This is agreed by our data, which had a median prescription isodose percentage of 45%. For CK, nonisocentric planning allows user to prescribe to a higher percentage of isodose line. In our CK plans, the median prescription isodose level reached 86% (range 77%‐92%), while a high level of coverage (median 98%, range 96%‐100%) was maintained. This level of coverage by CK was comparable to GK in all cases.

On average, eight isocentric shots per lesion were used in GK, while 75 beams per lesion were needed for a nonisocentric CK plan. The median values for minimum dose were 18.2 Gy and 17.6 Gy for GK and CK, respectively (p=0.40, not significant). The median values for mean dose were 29.6 Gy (GK) and 20.6 Gy (CK); for maximum doses, 40.3 Gy (GK) and 22.7 Gy (CK). The differences for both mean and maximum doses reached statistical significance (p<0.00001).

**Table 2:** Summary of tumor locations (26 lesions)

		n(%)
Frontal	Left	4 (15%)
	Right	4 (15%)
Temporal	Left	3 (11%)
	Right	0 (0%)
Parieto‐occipital	Left	1 (3.8%)
	Right	2 (7.7%)
Cerebellar	Left	3 (12%)
	Right	5 (19%)
Vermis		3 (12%)
Brain stem		1 (3.8%)

**Table 3:** Comparison of GK and CK plan parameters (26 lesions)

	*GK*	*CK*	
	*median (range)*	*median (range)*	p *value*
Planned tumor volume (cm^3^)	2.50 (0.044‐1.99)	2.49 (0.053‐20.0)	0.94
Prescription dose (Gy)	18.0 (15.0‐22.0)		N/A
Prescription isodose %	45 (40‐50)	86 (77‐92)	< 0.00001
GTV coverage (%)	100 (96‐100)	98(96‐100)	0.99
Minimum dose (Gy)	18.2 (12.8‐26.7)	17.6 (13.0‐21.4)	0.40
Mean dose (Gy)	29.6 (19.9‐38.5)	20.6 (16.1‐25.8)	< 0.00001
Maximum dose (Gy)	40.3 (29.4‐51.0)	22.7 (16.5‐28.6)	< 0.00001
Homogeneity index (HI)	2.22 (2.00‐2.50)	1.18 (1.09‐1.30)	< 0.00001
GK=Gamma Knife, CK=CyberKnife, N/A=not applicable, GTV=gross tumor volume.

The homogeneity index (HI) measures the ratio of maximum dose to prescription dose. Consequently, it often favors nonisocentric planning as employed by CK, as it yields a lower maximum dose and makes the overall plan more homogenous. HI is also inversely correlated to the prescription isodose percentage. The median values for HI were 2.22 and 1.18 for GK and CK plans, respectively (p<0.00001). One lesion was excluded from this series of conformality index (CI) analyses (one patient had a central brainstem metastasis and did not tolerate whole‐brain radiotherapy). He was given a palliative dose by GK, and his lesion was not covered entirely (coverage 82% only).

Table 4 summarizes the various conformity indices applied in this study. From “loose” to “rigorous,” these indices may be ranked in this order: RTOG_CI, CI, and nCI. As more than 80% of evaluable lesions reached 100% coverage in GK plans, the three GK‐related indices all had a median value of 1.76. For CK, an isodose line which yielded a coverage value of 97% was typically prescribed. From RTOG_CI to nCI, the CI values slowly increased (becoming less conformal), as coverage was taken into consideration. As a result, the CI and nCI comparisons were statistically insignificant (p≥0.05), while the RTOG_CI index did reach statistical significance (p=0.022). Adjusted for coverage, the CK's conformity advantage diminished and became negligible in CI and nCI.

Averaged volume ratio (AVR) and gradient index (GI) methods were calculated to evaluate dose falloff, which was commonly used in comparing various SRS modalities.^(18)^ For both AVR and GI, the calculated percentages were normalized with respect to the prescription dose. With the same prescription dose, CK prescribes to a higher isodose line percentage. For example, an equivalent plan may prescribe 20 Gy to an 80% isodose line in CK, versus 50% isodose line in GK. The normalized 90%, 80%, 60%, 50%, 40%, 20%, and 10% isodose lines were calculated. Table 5 shows the AVR of different isodose volumes in relation to the prescribed isodose volume.

**Table 4:** Summary of three comparative conformity indices

	*GK*	*CK*	
	*median (range)*	*median (range)*	p *value*
RTOG CI	1.76 (1.12‐4.14)	1.53 (1.16‐2.12)	0.022
CI	1.76 (1.15‐4.14)	1.55 (1.18‐2.21)	0.050
nCI	1.76 (1.59‐4.14)	1.57 (1.20‐2.30)	0.082

a GK=Gamma Knife; CK=CyberKnife; RTOG CI=Radiation Therapy Oncology Group conformity index; CI=conformity index; nCI=new conformity index.

**Table 5:** Summary of GK and CK peripheral dose falloff

	*Gamma Knife SRS*		*Cyber Knife SRS*		
*Percent* ^a^	*median (range)*	*AVR*	*median (range)*	*AVR*	p *value*
[GTV]	2.50 (0.04‐19.9)	0.57	2.49 (0.05‐20.0)	0.66	0.94
100	4.40 (0.20‐37.8)	1	3.75 (0.08‐42.3)	1	N/A
90	5.35 (0.22‐46.0)	1.21	5.62 (0.13‐81.5)	1.55	<0.00001
80	6.40 (0.28‐56.7)	1.48	7.69 (0.22‐112.2)	2.15	<0.00001
60	9.75 (0.44‐88.3)	2.30	12.8 (0.57‐173.7)	3.80	<0.00001
50	12.9 (0.58‐110.1)	2.99	17.3 (0.90‐213.0)	5.21	<0.00001
40	17.0 (0.83‐133.4)	4.05	25.2 (1.54‐264.9)	7.59	<0.00001
20	35.8 (2.80‐172.7)	8.03	80.9 (5.56‐522.3)	23.49	<0.00001
10	49.6 (5.90‐178.7)	11.05	183.3 (14.4‐1058.2)	64.88	<0.00001

a ^a^ Percent refers to normalized levels (compared with 100) in relation to prescription dose, not actual isodose line percentage.

SRS=stereotactic radiosurgery; GTV=gross tumor volume; AVR=averaged volume radio; N/A=not applicable.

As isodose line percentage decreases, more normal tissue will be included in the irradiating volume. In CK, a slower rate of falloff was observed. This difference appeared more significant at lower normalized isodose levels. For example, at normalized 20% isodose line, AVR was found to be 64.88 in CK (versus 8.03 in GK). Compared to GK, 15.46 more times of PIV were included by CK at this level. These differences were statistically significant across all levels (for normalized 90%, 80%, 60%, 50%, 40%, 25%, 20%, and 10%, p<0.00001).

Table 6 summarizes the results of V12, V12net, GI50, and GI25. Similarly, GK generated better plans compared with CK. GI50 and GI25 both reached statistical significance (p<0.00001). There was a trend of smaller V12 and V12net volumes favoring GK. Additionally, a wide range of indices was noted in CK. For example, there is a wider range for CK's GI50 (3.42‐11.68) compared to GK (2.48‐3.67). This effect was likely multifactorial, and may not be generalizable. The median “beams‐on” times for GK and CK (extrapolated from monitoring unit) were 107 and 220 minutes, respectively.

**Table 6:** Summary of GK and CK peripheral dose falloff

	*GK*	*CK*	
	*median (range)*	*median (range)*	p *value*
V12 (cm^3^)	7.09 (0.44‐64.60)	11.12 (0.57‐127.5)	0.13
V12net (cm^3^)	5.04 (0.40‐44.70)	8.64 (0.52‐107.7)	0.077
GI50	2.91 (2.48‐3.67)	4.90 (3.42‐11.68)	<0.00001
GI25	6.58 (4.18‐10.20)	14.85 (8.80‐48.37)	<0.00001

a V12=volume covered by 12 Gy isodose; V12net=volume covered by 12 Gy isodose,excluding gross tumor volume; GI=gradient index.

## IV. DISCUSSION

Recently, a German study^(15)^ reported a matched‐pair analysis between 423 GK and 73 CK patients with single brain metastases. Compared to GK, the authors reported significantly lower numbers strongly favoring CK, including minimum dose, maximum dose, isodose line percentage used, PIV, CI (equivalent to RTOG_CI), HI, V10, and V10net. In contrast, we did not find a significant difference in minimum dose in our study. Also, our V12 and V12net (analogous to V10 and V10net) were higher in CK, not lower. A different range of CK isodose prescription percentage was used in the German study (67%±5%) vs. ours (85%±4%), which may account for some of the differences observed. All other dosimetric results and findings were similar or the same.

Investigators have previously examined results from multicenter randomized trials which involved stereotactic radiosurgery as a boost. However, these conclusions were limited to subgroup analyses. In the RTOG 9005 final report, the authors found that patients treated with linac had a 2.84 times higher risk of local tumor progression, as compared to patients being treated with GK. This observation led them to suspect that “(GK) may have effectively boosted the central, hypoxic, more radioresistant portion of the tumor, accounting for the better local control ... one possible explanation lies in the inherent inhomogeneity that exists in the dosimetry of GK radiosurgery”.^(19)^ However, this was not seen in a later trial (RTOG 9508), which also included SRS boosts. No significant difference was observed in progression‐free survival between GK and linac choices.^(20)^ A multi‐institutional analysis of 502 patients^(21)^ also similarly concluded that GK versus linac did not seem to matter — a SRS boost increased median survival, regardless of modality choice, compared with patients only treated with WBRT alone (16.1, 10.3, and 8.7 vs. 7.1, 4.2, and 2.3 months for RpA classes I, II, and III, respectively, p<0.05). CK was not included as a SRS option, as it is a relatively new modality.

Through measurement of various indices, CK appeared to produce more conformal plans in our series as compared to GK. The difference in RTOG_CI index was statistically significant. Modified (CI) and new conformality (nCI) indices barely missed statistical significance; however, due to limited sample size, the post hoc statistical power was low (47.2%). Some studies showed that SRS dosimetric conformality may relate to clinical outcome. For example, according to the Stanford experience with resected cavities,^(12)^ the authors observed that higher conformality indices correlated with lower rate of local tumor recurrence. As a result, they recommended the use of a 2 mm margin when treating brain metastases postoperatively.

Regardless of modality choice, an important motivation for optimizing gradient index is to prevent SRS complications. In the RTOG 9005 study, higher rates of CNS toxicity were noted in patients with larger size of tumors, which was the most important predictor for radionecrosis.^(19)^ Other risk factors included increased volume receiving 10 Gy or more, higher radiation dose, repeated radiosurgical treatments to the same tumor, and increased size of erroneously irradiated normal brain tissue (i.e., a less conformal plan).^22^, ^23^, ^24^ For GK, tissue volume enclosed by the 12 Gy isodose line also correctly predicted complication risk in patients with AVMs and other non‐AVM intracranial tumors.^25^, ^26^ Our results showed significantly different falloff profiles between GK and CK, as evident in gradient index, AVR, V12, and V12net calculations. Recently, a joint study by UCSF and Princess Margaret Hospital (Canada) observed nearly identical dose falloff profiles among the GK, CK, and Novalis systems.^(18)^ While our data matched well to that of their GK system, significant GI difference was observed in the CK series. The UCSF researchers noted a GI of 2.88±0.82 with a prescription range of 49‐78% (n=10), while in our series, a GI of 4.95±0.91 was observed with a prescription range of 77‐92% (n=26). We suspected that the use of a higher prescription isodose percentage may account for this difference. A more fundamental understanding of CK dosimetric properties will be needed in the future.

Compared to GK, CK's GI50 was poorer in our series. Two other studies from University of Southern California also observed that CK had a slower falloff as compared with GK.^(27)^ However, in absolute scale, this difference was small and may not be clinically significant. For a hypothetical 2 cm diameter spherical target, the additional falloff distance as incurred by CK is only 2.6 mm in any direction at GI50. Magnitudes of V12 and V12net (or V10/V10net) correlated with chance of developing radionecrosis, an uncommon but feared complication of SRS.^22^, ^24^, ^26^ V12 and V12net values are a function of homogeneity, conformity, prescription dose, and peripheral falloff. In our series, GK appeared slightly better, but it did not reach statistical significance. Our study has the following limitations: 1) did not address hypofractionation, which is possible for both modalities but more cumbersome for GK due to headframe immobilization; 2) was not designed to examine the clinical outcome and long‐term control of brain metastases by various SRS modalities; 3) was not planned for analyzing the influence of patient and tumor characteristics on the conformality of the treatment modalities.

## V. CONCLUSIONS

In patients with brain metastases, we showed that CK can create dosimetrically equivalent plans, as compared to GK. With similar coverage and minimum dose, both modalities effectively irradiated the entire tumor volume. Compared to GK, CK produced more homogenous plans with statistically lower mean and maximum doses. There is also a trend for CK being more conformal by RTOG_CI, CI, and nCI indices, with RTOG_CI reaching statistical significance. GK had a faster rate of dose falloff to the periphery in our series, as suggested by AVRs, V12, V12net, and GI. We suspect that this may due to a higher prescription isodose percentage in our planning with CK; further dosimetric investigation is warranted. Our results showed that ideal conformity and dose falloff may not always be easily and simultaneously achieved, which call for further investigation. For example, a combined index taking into account of both conformity and dose gradient effect has recently been proposed.^(17)^

In this project, we built a dosimetric foundation for systematically comparing various SRS modalities, which may be correlated with clinical outcome when combined with future studies. Our study provided preliminary insight in guiding future research and interdepartmental collaboration in considering whether GK or CK may be more suitable for the individual cancer patient with disabling brain metastases. Other factors, such as number and location of these lesions, patient's preference for SRS and also whole brain radiotherapy, personal history of previous intracranial irradiation, and functional status of the patient, will also need to be considered. Future work may include multimodality dosimetric comparison, and also a detailed economic analysis in comparing GK, CK, a linac‐based system with other emerging technologies such as RapidArc,^(28)^ and proton therapies.

## Figures and Tables

**Figure 2:**
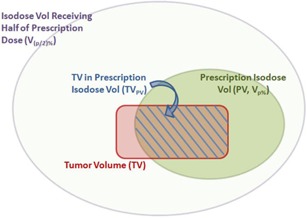
A graphical presentation of dosimetric nomenclatures.

## Appendix 1: Technical Summary of Conformity and Gradient Indices

This session briefly summarizes the theories and rationale of using dosimetric indices in comparison of rival SRS plans. It involves the construction and application of two major concepts: conformity and gradient indices.

Delivering conformal irradiation is a cornerstone of good radiotherapy practice. Medical physicists developed clinical and physical techniques in ensuring that the planned volume receiving the prescription dose can be best geometrically shaped in adapting to the target or tumor volume delineated on CT or MR imaging. This is especially important for stereotactic radiosurgery, for both ensuring accurate and efficacious radiation delivery (“hit the target”) and protecting the surrounding healthy tissues from excessive radiation injury (“do not harm the innocent”). The goal of stereotactic radiosurgery is to deliver extremely focused radiation beams to the chosen target, with submillimeter accuracy. It is possible to capture and compare the level of conformity quantitatively. In 1993, The Radiation Therapy Oncology Group (RTOG) proposed the first version of conformity index (also called the “RTOG CI”):

(1) CIRTOG=PVTV

where *PV* is the total tissue volume which receives the prescription isodose, and *TV* is the actual tumor volume (equals to GTV when no additional margin is added). If ${\text{CI}}_{\text{RTOG}}$ is greater than 1, the prescription isodose line includes healthy tissue other than the tumor volume. If ${\text{CI}}_{\text{RTOG}}$ is less than 1, the tumor volume is under‐irradiated. By RTOG standards, a treatment plan is acceptable if CI is between 1 and 2. A conformity index between 2 and 2.5, or 0.9 and 1, is a “minor” violation; an index less than 0.9 or more than 2.5 is considered a “major” violation. Researchers, however, soon discovered that this definition failed to take into account the level of overlapping, or spatial interaction, of the two volumes involved. For example, two identical volumes (PV, TV) which do not overlap with each other at all (i.e., target is entirely missed) will still receive a CI of 1, a number misleadingly indicating perfect conformation. It then became clear that information regarding coverage must be integrated. Here, coverage (CO) is volumetrically defined as

(2) CO(Coverage)=TVPVTV×100%

where $TV_{PV}$ is the volume of the tumor receiving at least the prescription dose or more. Traditionally, coverage is expressed in percentage. In order to accurately account for target coverage in conformity evaluation, a new index called healthy tissue conformity index (HTCI) was proposed by the Lomax and Scheib group.^(29)^ The inverse of this index is reported by the CyberKnife's MultiPlan. This CI is defined as:

(3) CI=PVTVPV

which is sometimes also called the modified conformity index (mCI). However, this conformity index cannot detect under‐treatment (e.g., a prescription volume to 1 cm sphere completely included in a 2 cm TV sphere will produce a deceptively perfect CI of 1, yet the coverage is only 12.5% by volume). A new CI was then used by CyberKnife, which could correct for this problem (the new CI can also be easily adapted to other stereotactic systems as well). This new CI may represent an improvement to the CI as defined in Eq.(3) because it balances the proportion of the TV covered by the PV (${\text{TV}}_{\text{PV}}/\text{TV}$, an “under‐treatment” ratio), with the proportion of the PV inside the tumor volume (${\text{TV}}_{\text{PV}}/\text{PV}$, an “over‐treatment” ratio). The new CI can then be calculated as:

(4) nCI=(PV⋅TV)PVTV2

Here, the nCI is essentially the reciprocal product of the popular Ian Paddick's index.^(17)^

An algebraic examination reveals that the three conformality indices above can be inter‐converted through calculation of the coverage factor (Eq. (5)), which is a piece of information readily available in the normal tissue dose‐volume histogram (DVH). This becomes the basis for numerical conversion, as GammaPlan does not automatically calculate CI or nCI. In particular, when coverage equals 100% (which were the majority of brain metastasis cases in GammaPlan), the three indices essentially become equal in value. This relationship can then be summarized as:

(5) nCI=CI×(1CO)=CIRTOG×(1CO)2

Finally, a volume‐based gradient index (GI) can also give us more information about the quality of a SRS plan. An important goal of stereotactic radiosurgery is to ensure that the surrounding tissue gets as little‐to‐no radiation dose as possible (i.e., a rapid gradient of dose falloff must be created at the margin of the prescription isodose line). Ideally, all radiation should be contained within the prescription volume, and nothing else outside of the PV to periphery (i.e., no radiation “spilling”). A gradient index (GI)^(17)^ provides a convenient method in measuring this intrinsic property of a SRS plan, which is independent of tumor volume and shape. The formula is:

(6) GI50=V(p/2)%Vp%

where $V_{p\%}$ is the tissue volume enclosed by the prescription isodose line, and $V_{(p/2)\%}$ is the volume enclosed by half (50%) of the prescribed dose. The advantages of using Eq. (6) are that: 1) these volumes are easily extractable from the DVH; and 2) it allows fair comparison across different rival plans which may not prescribe to same percentage of isodose lines. An optimal plan should enclose small volume even at their lower isodose lines. An analogous gradient index $\left({\text{GI}}_{25}\right)$ at 25% prescription isodose line can then also be defined. ${\text{GI}}_{25}$ is the ratio of $V_{(p/4)\%}$, the volume enclosed by one fourth of the prescription dose to $V_{p\%}$.

The homogeneity index (HI) is also a common measure for comparing rival SRS plans. HI is the ratio of the maximum dose to the prescription dose (maximum dose is always at 100% isodose in both CK and GK planning). Figure 2 gives a pictorial illustration of the dosimetric volumes as utilized in this project.
